# Sorafenib Combined with Transarterial Chemoembolization versus Transarterial Chemoembolization Alone for Advanced-Stage Hepatocellular Carcinoma: A Propensity Score Matching Study

**DOI:** 10.1371/journal.pone.0096620

**Published:** 2014-05-09

**Authors:** Hao Hu, Zhenhua Duan, Xiaoran Long, Yancu Hertzanu, Haibin Shi, Sheng Liu, Zhengqiang Yang

**Affiliations:** 1 Department of Interventional Radiology, The First Affiliated Hospital of Nanjing Medical University, Nanjing, China; 2 Chengdu Center for Disease Control and Prevention, Chengdu, China; 3 School of Pharmacy, Anhui Medical University, Hefei, China; 4 Faculty of Health Sciences, Ben-Gurion University of the Negev, Beer-Sheva, Israel; Northwestern University Feinberg School of Medicine, United States of America

## Abstract

**Aims:**

The purpose of the present study was to compare the efficacies of transarterial chemoembolization (TACE) combined with sorafenib versus TACE monotherapy for treating patients with advanced hepatocellular carcinoma (HCC).

**Methods:**

We enrolled 321 patients and selected 280 with advanced HCC (Barcelona Clinic Liver Cancer stage C) who underwent TACE therapy between February 2009 and February 2013. TACE alone (monotherapy group) was administered to 198 patients (70.7%), and the remaining 82 (29.3%) underwent repeat combined TACE and sorafenib therapy (combined group). To minimize selection bias, these latter 82 patients were matched using propensity-score matching at a 1∶2 ratio with 164 patients who received TACE monotherapy. The primary endpoints were overall survival (OS) and related subgroup analysis. The secondary endpoints were time to progression (TTP) and treatment-related adverse events.

**Results:**

Of the respective patients in the combined and monotherapy groups, 64.6% and 49.2% had vascular invasion, 87.8% and 91.1% had extrahepatic metastasis, and 54.3% and 47.1% had both. In the propensity-score–matched cohort, the OS survival of the combined group was significantly higher compared with the monotherapy group (7.0 months vs. 4.9 months, respectively, *P* = 0.003). The TTP was significantly longer in the combined group (2.6 months vs. 1.9 months, respectively, *P* = 0.001). Subgroup analysis showed that the outcomes of patients with advanced HCC without main portal vein invasion who were treated with combined therapy were significantly better compared with those who received monotherapy (*P*<0.05). Univariate and subsequent multivariate analyses revealed that the addition of sorafenib was an independent predictor of favorable OS and TTP (adjusted hazard ratios, 0.63 and 0.62, respectively; *P*<0.05 for both).

**Conclusion:**

Sorafenib plus TACE was more effective than TACE monotherapy for treating patients with advanced HCC without main portal vein invasion. Future trials with larger samples are required to validate these preliminary findings.

## Introduction

Hepatocellular carcinoma (HCC) is the third leading cause of cancer death and the fifth most common cancer worldwide with increasing incidence with more than 500,000 new cases reported each year [1], [2]. Despite recent improvements in surveillance programs, a high percentage of patients with HCC, regardless of geographical location or socioeconomic status, are not diagnosed until an advanced stage that is characterized by vascular invasion and distant metastasis that corresponds to Barcelona Clinic Liver Cancer (BCLC) stage C (portal invasion, N1, M1, PS1-2).

Transarterial chemoembolization (TACE) is one of the most commonly used treatments for unresectable HCC. Current guidelines recommend TACE as the standard treatment of BCLC-B patients [3]. TACE prolongs 2-year survival by 63% compared with 27% achieved with supportive care [4]. Recent studies show that TACE is effective for controlling symptoms of patients with advanced HCC, including those with vascular invasion or metastases, and is a common mainstay palliative modality in Asia [5]–[8]. In addition, in developing countries, such as China, economic conditions restrict the application of sorafenib in some patients. Therefore, consecutive TACE is still used to treat selective patients with portal vein thrombosis (PVT).

Sorafenib, which is an orally administered small molecule, inhibits multiple protein kinases. At present, sorafenib is the only approved systemic therapy for patients with advanced stage (BCLC-C) [9], and phase III randomized clinical trials demonstrate that it is efficacious for prolonging time-to-progression (TTP) and median survival of patients with HCC [10], [11]. However, it displays only modest clinical efficacy as a single therapy for this poorly controllable disease; therefore, new treatment strategies are urgently required [11], [12]. One such possibility is suggested by the encouraging results of recent phase II trials that evaluated concurrent treatment of patients with advanced unresectable HCC with TACE and sorafenib [13], [14]. Therefore, sorafenib combined with TACE is now more widely applied to treat unresectable HCC.

The results of clinical studies completed in the United States, Italy, Korea, China, and Japan are inconsistent. Further, BCLC stages B and C include a spectrum of patient groups with various diseases. This heterogeneity precluded a more detailed subgroup analysis for patients with different extents of disease. Furthermore, patients with BCLC stage C disease presented with different clinical manifestation, including portal vein invasion and tumor extrahepatic metastasis (TEM). No other evidence shows that combined therapy enhances the survival rate of patients with variations in the characteristics of BCLC stage C.

The main goal of the present study was to compare the overall survival (OS) and TTP of patients with advanced HCC who were treated with sorafenib combined with TACE compared with TACE monotherapy. We further evaluated the OS of patients within different BCLC stage C subgroups treated with these same regimens.

## Materials and Methods

### Study Design

**Figure 1:**
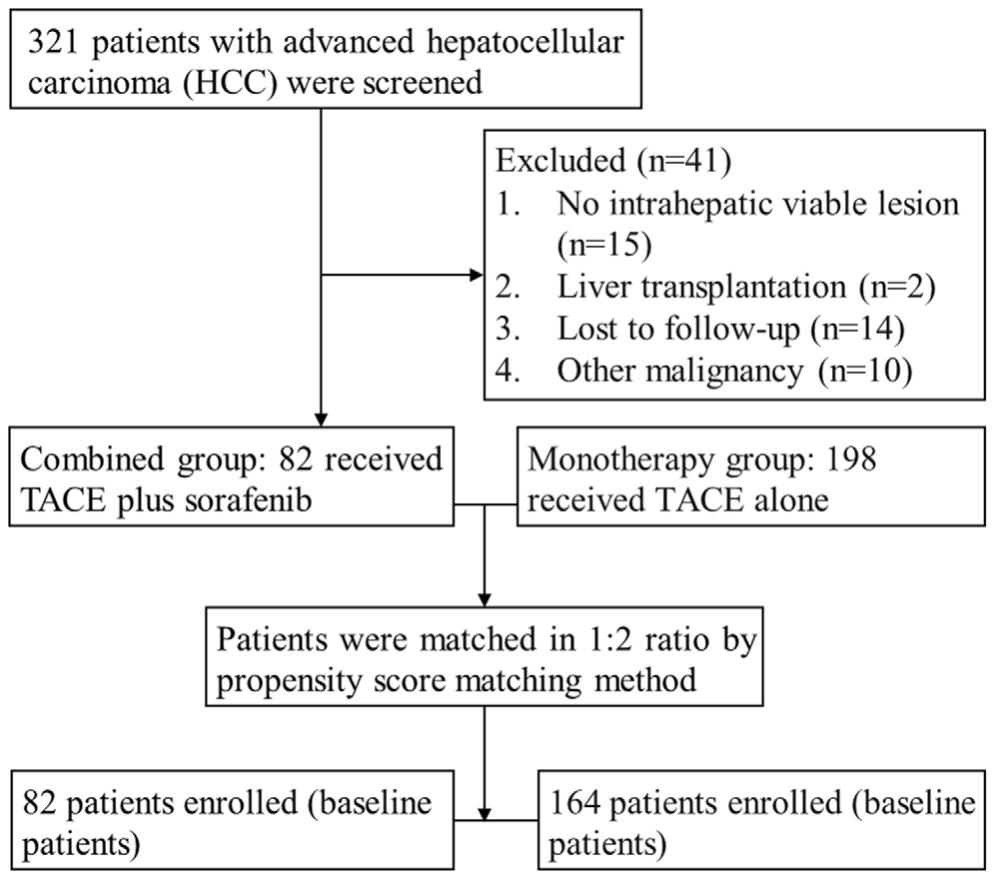
Patient selection.

We conducted a retrospective cohort study that enrolled 321 consecutive patients with advanced HCC who were admitted to our department from February 2009 to November 2011 (Figure 1). 41 patients were excluded in the study. The remaining patients were divided into two groups, each of which received sorafenib plus TACE (combined therapy group) or TACE monotherapy (monotherapy group). The Institutional Review Board of Jiangsu Province People's Hospital (Nanjing Medical University, Nanjing, Jiangsu Province, China) approved this study. The duration of patients' survival was calculated from the date of recruitment to death or study closure, and TTP was calculated from the date of recruitment to radiological progression. Follow-up was terminated upon death of a patient or on February 28, 2013.

### Evaluation of Outcomes

OS was the primary endpoint of the analysis, which was defined as the time from enrollment to death from any cause or to the last follow-up in censored patients. The secondary endpoints were TTP based on the modified Response Evaluation Criteria in Solid Tumors (mRECIST) and treatment-related adverse events (AE). Treatment-related AE were assessed using the Common Terminology Criteria for AE (CTCAE) version 4.0.

### Patient Selection Criteria

HCC was reconfirmed in all patients based on the practice guidelines of the American Association for the Study of Liver Disease [15]. Inclusion criteria were as follows: (1) patients classified with BCLC stage C disease, which is generally not considered an indication for curative-intent treatment; (2) Eastern Cooperative Oncology Group performance status 0–2; (3) vascular invasion and/or extrahepatic spread; (4) patients with Child's A and B cirrhosis; and (5) patients treated with TACE plus sorafenib or TACE monotherapy. Exclusion criteria were as follows: (1) patients who received liver transplants at any time; (2) patients with only nodal or distant metastases without viable lesions in the liver; (3) patients with secondary malignancies; (4) patients with a history of concomitant use of some other targeting agent, chemotherapy and immunotherapy; and (5) patients who were lost to follow-up. All patients were informed of the advantages and disadvantages of the two treatment options, including treatment outcomes, treatment-related morbidities, and costs; and the final treatment decision was made jointly by the physician and patient based on fully respecting the patient's willingness to participate [16].

### Treatment

#### TACE

Angiography was performed using a 5-Fr catheter inserted through the femoral artery with selection for the hepatic or superior mesenteric artery based on tumor arterial blood supply, which was confirmed using arteriography. Guided by fluoroscopy, the tip of the catheter was superselected into the tumor-feeding branches (a microcatheter was used if necessary). After identification of the target artery in the tumor, chemoembolization was achieved as selectively as possible for all targeted lesions in left and right lobes of the liver with 2–20 mL of an emulsion consisting of a 1∶1 ratio of cisplatin and iodized oil depending on liver function and tumor size. Gelatin sponge or polyvinyl alcohol particles were injected to embolize tumor-feeding arterioles if necessary until there was no longer any tumor staining after repeat angiography. In patients with tumor thrombosis in the main portal branch and/or Child–Pugh class B liver function, only chemolipiodolization without gelatin sponge particles was performed because of concerns about deterioration of hepatic function after arterial embolization. Patients were subsequently admitted for the management of potential postembolization syndrome. Dynamic liver computed tomography (CT) or magnetic resonance (MR) imaging was performed 6–8 weeks after the procedure to detect lipiodol retention within the tumor and residual viable tumor tissue. When residual viable tumors were confirmed or new lesions developed in patients with adequate liver function, repeated TACE procedures were performed.

#### Sorafenib treatment

All patients were given detailed information regarding sorafenib treatment, including its efficacy and potential adverse effects. Patients decided on whether to undergo sorafenib or combined treatment. All patients who chose combined therapy received oral sorafenib (400 mg) twice daily after TACE, except for those who developed a contraindication to sorafenib (e.g. insufficient liver function). For patients who chose TACE plus sorafenib therapy, the efficacy of combined treatment was assessed using dynamic CT or MR imaging of the liver 6–8 weeks after treatment. Total bilirubin and alanine aminotransferase (ALT) levels were determined post-TACE. To ensure patients' safety, the dose of sorafenib was reduced, or treatment was delayed or temporarily discontinued when we observed clinically significant toxicity (≥ grade 3) based on the National Cancer Institute's CTCAE version 4.0 or at the physician's discretion. Treatment using dose escalation or rechallenge with sorafenib was decided when toxicity decreased and the patient tolerated the medication. During follow-up, the levels of aspartate aminotransferase (AST), ALT, α-fetoprotein (AFP), bilirubin, albumin, and prothrombin time were determined every 4 weeks to evaluate liver function. Meanwhile, dynamic liver CT or MR imaging was performed every 6–8 weeks after treatment to evaluate response.

### Statistical Analysis

Propensity score analysis was performed to adjust for potential bias and is used often in observational studies because of nonrandomized group assignment [17]. A propensity score for each patient was calculated using multivariable logistic regression. The covariates included in the analysis were as follows: gender, age, viral hepatitis, tumor metastasis, previous treatment of a tumor, ascites, Child–Pugh score, liver cirrhosis, tumor phenotype, and serum AFP level. We used single nearest-neighbor matching with no replacement (a single participant could not be selected multiple times) to match patients in the TACE cohort to those receiving combined therapy (using Stata command psmatch2; StataCorp LP, College Station, TX, USA) [18]. Differences in baseline characteristics of patients of the two groups were compared using Fisher's exact or chi-square tests for categorical variables and a *t* test for continuous variables. To evaluate the benefit of combined therapy, we compared OS and TTP between propensity-score-matched cohorts. The Kaplan–Meier method was used to evaluate the effect of patient characteristics on OS and TTP. The significance of differences between groups was evaluated using the log-rank test. Univariate and multivariable Cox proportional hazards models were used to determine the effect of combined therapy on OS and TTP after adjusting for the prognostic variables, and hazard ratios (HRs) with 95% confidence intervals (CIs) were calculated. All tests were 2-sided. *P*<0.05 was considered statistically significant. All analyses were conducted using SPSS software package version 13.0 (SPSS, Chicago, IL, USA) and STATA 12.0 (StataCorp LP).

## Results

A total of 321 patients with advanced HCC were admitted to our department from February 2009 to November 2011. Forty one patients were excluded from this study using the criteria shown in Figure 1. Additional information is provided in Appendix S1. After filtering, 82 (29.3%) patients were treated simultaneously with at least one session of TACE at the beginning of sorafenib therapy (combined group) and 198 (70.7%) received TACE monotherapy (monotherapy group). In the combined group, the maximum number of TACE sessions per patient was 8 (average of 1.8 sessions per patient). Sorafenib treatment was initiated for 74 patients (90.2%) within 7 days after the TACE procedure (range 4–7 days). The treatment of the remaining 8 patients (9.8%) was delayed because of TACE-induced adverse effects, but all received sorafenib therapy within 14 days (range 8–14 days) after the completion of TACE. Sorafenib was administered to 29 of 82 (35.4%) patients in the combined group at a starting daily dose of 400 mg. The other 53 patients were treated with the full dose. Dose reduction or interruption (excluding routine interruption around the time of TACE) was required for 38 of the 82 patients in the combined group (46.3%). Treatment of 16 patients was interrupted.

After matching using the nearest available neighbor method (1∶2) based on the number of patients who accepted sorafenib, 164 patients treated with TACE monotherapy were matched for the analyses (Figure 1). The characteristics of the patients are listed in Table 1. After propensity-score matching, there were no significant differences among the baseline characteristics of the patients in the two groups with the exception of ascites. The median follow-up for all patients was 6.9 months (range, 1.2–37.4 months).

**Table 1:** Baseline characteristics of patients before and after propensity-score matching.

Variables	Combined group (n = 82)	Monotherapy group (pre-match, n = 198)	P value	Monotherapy group (matched, n = 164)	P value
Sex, n (%)			0.03		0.80
Male	69 (84.1)	159 (80.3)		140 (85.4)	
Female	13 (15.9)	39 (19.7)		24 (14.6)	
Age					
All patients	61±11	57±12		60±11	
≥60 years	48 (58.5)	79 (40.1)	0.07	72 (43.9)	0.72
<60 years	34 (41.5)	119 (59.9)		92 (56.1)	
Viral hepatitis, n (%)			0.34		0.83
HBV	68 (82.9)	158 (79.8)		139 (84.8)	
HCV	6 (7.3)	10 (5.1)		10 (6.1)	
No infections	8 (9.8)	30 (15.1)		15 (9.1)	
Tumor metastasis, n (%)			0.51		0.81
Main portal vein thrombosis	20 (24.4)	39 (19.7)		35 (21.3)	
Portal vein branch thrombosis	21 (25.6)	52 (26.4)		45 (27.4)	
Distant tumor metastasis	27 (32.9)	60 (30.3)		49 (29.9)	
Portal vein thrombosis and distant tumor metastasis	14 (17.1)	47 (23.6)		35 (21.4)	
Previous tumor treatment, n(%)			0.01		0.72
Yes	43 (52.4)	117 (59.1)		90 (54.9)	
No	39 (47.6)	81 (40.9)		74 (45.1)	
Ascites, n (%)			0.031		0.03
Absence	29 (35.4)	49 (24.6)		35 (21.4)	
Small amount	33 (40.2)	101 (50.9)		83 (50.6)	
Moderate amount	16 (19.5)	45 (22.8)		44 (26.8)	
Large amount	4 (4.9)	3 (1.7)		2 (1.2)	
Child–Pugh score, n (%)			0.53		0.22
A	58 (70.7)	134 (67.5)		103 (62.8)	
B	24 (29.3)	64 (32.5)		61 (37.2)	
Liver cirrhosis, n (%)	58 (70.7)	151 (76.5)	0.24	127 (77.4)	0.25
Type of tumor, n (%)			0.12		0.24
Nodular	39 (47.6)	90 (45.3)		65 (39.6)	
Infiltrative	43 (52.4)	108 (54.7)		99 (60.4)	
Serum AFP ≥ 400 ng/mL, n (%)	55 (67.1)	138 (69.6)	0.28	119 (72.6)	0.28

TACE, transarterial chemoembolization; BCLC, Barcelona Clinic Liver Cancer; HBV, hepatitis B virus; HCV, hepatitis C virus.

### OS Analysis

**Figure 2:**
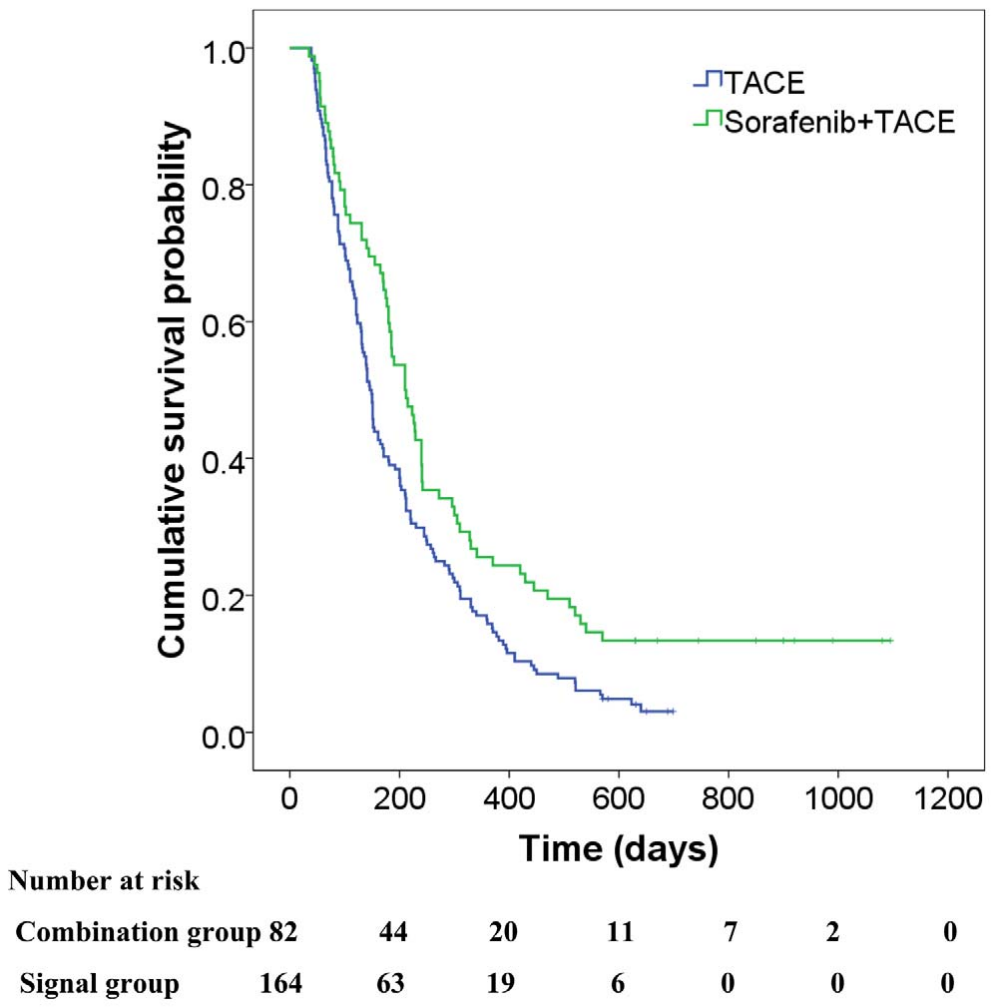
Kaplan–Meier analysis of OS of the combined and monotherapy groups. The Kaplan–Meier curves show the OS of the combined and monotherapy groups for propensity-matched patients. The median OS of the combined group was significantly longer compared with the monotherapy group in the matched model (7.0 months vs 4.9 months, respectively; *P* =  0.003).

During follow-up, 229 of the 246 patients (93.1%) (71 of 82 patients (86.6%) in the combined group and 158 of 164 patients (96.3%) in the monotherapy group) died because of HCC progression. The median OS was significantly longer in the combined group compared with the monotherapy group (7.0 months vs. 4.9 months, respectively; *P* = 0.003) (Figure 2). Unadjusted and adjusted HRs and CIs for evaluating the effect of treatment regimen on OS were 0.65 (95% CI, 0.49, 0.87; *P* = 0.0003) and 0.63 (95% CI, 0.48, 0.84; *P* = 0.002), respectively (Table 2). Covariates independently associated with OS were tumor metastasis, ascites, Child–Pugh class, and serum AFP level (*P*<0.05 for each; Table 2).

**Table 2:** Cox Regression Model Analysis of Unadjusted and Adjusted HR.

Variables	OS: Univariate Analysis		OS: Multivariate Analysis		TTP: Univariate Analysis		TTP: Multivariate Analysis	
	HR	P Value	HR	P Value	HR	P Value	HR	P Value
Sorafenib	0.65(0.49, 0.87)	0.0003	0.63(0.48,0.84)	0.002	0.62(0.47,0.82)	0.001	–	–
Age-group	0.98(0.76,1.28)	0.91	–	–	1.06(0.82,1.37)	0.66	–	–
Sex	0.79(0.74,1.50)	0.79	–	–	1.13(0.80,1.61)	0.49	–	–
Viral hepatitis	0.92(0.68,1.26)	0.61	–	–	1.01(0.76,1.34)	0.962	–	–
Main portal vein invasion	1.05(0.72,1.55)	0.79	–	–	1.03(0.70,1.52)	0.888	–	–
Portal vein branch invasion	0.44(0.30,0.66)	<0.0001	1.30(0.83,2.04)	0.25	0.36(0.24,0.54)	0	1.02(0.66,1.56)	0.947
Distant tumor metastasis	0.56(0.38,0.81)	0.002	2.02(1.29,3.16)	0.002	0.38(0.26,0.56)	0	1.21(0.79,1.87)	0.384
Previous treatment of tumor	0.84(0.65,1.09)	0.18	–	–	0.78(0.61,1.01)	0.056	–	–
Ascites	2.79(2.31,3.37)	0	2.57(1.97,3.35)	0	3.19(2.63,3.88)	0	2.40(1.84,3.13)	0
Child–Pugh score	3.52(2.60,4.75)	0	2.22(1.52,3.25)	0	4.43(3.25,6.05)	0	2.55(1.75,3.72)	0
Liver cirrhosis	1.31(0.98.1.77)	0.072	–	–	1.32(1.01,1.76)	0.043	1.22(1.03,1.64)	0.006
Type of tumor	1.43(1.09,1.86)	0.009	1.16(0.87,1.54)	0.302	1.54(1.19,2.01)	0.001	1.19(0.90,1.57)	0.22
Serum AFP level	3.22(2.30,4.50)	0	1.98(1.35,2.89)	0	3.34(2.41,4.64)	0	2.18(1.49,3.21)	0

### Subgroup Analysis of the Combined Group

**Figure 3:**
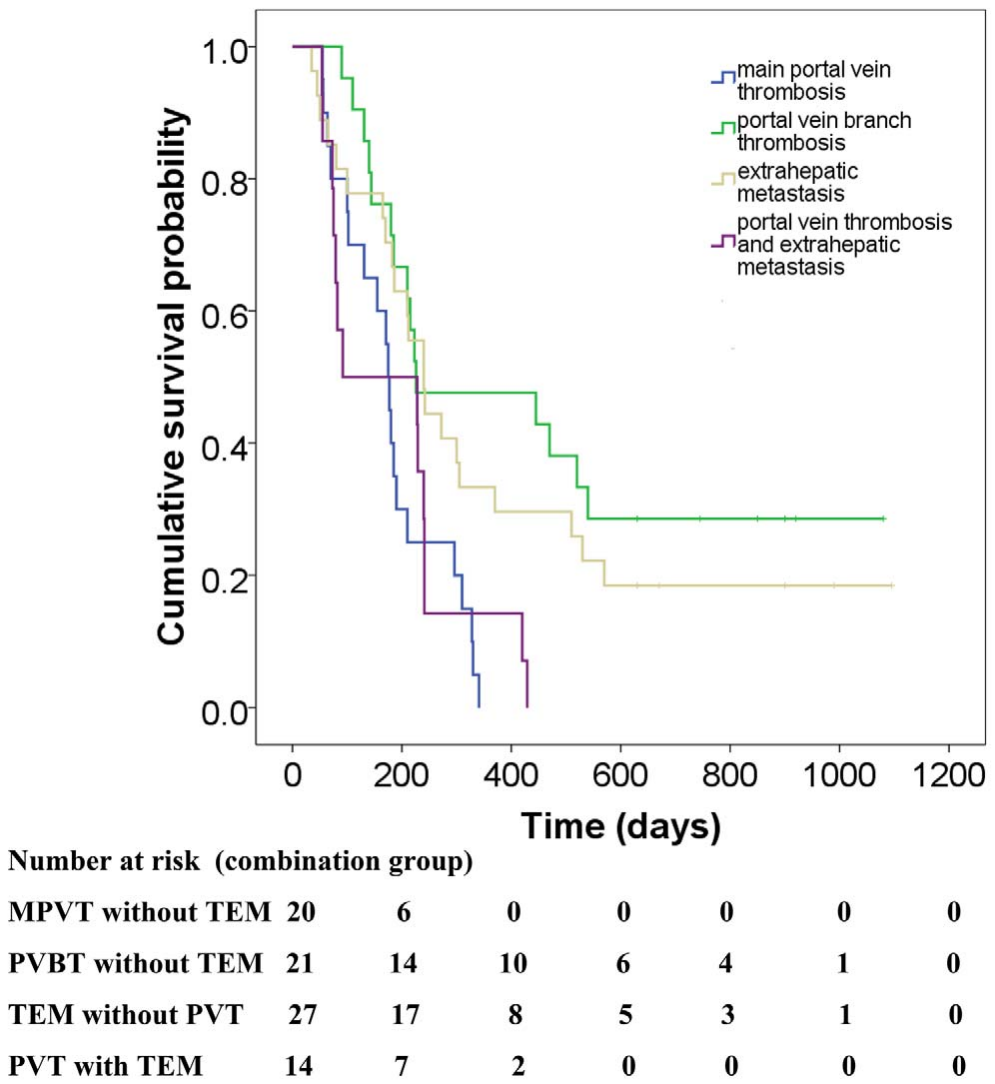
Kaplan–Meier analysis of overall survival of subgroups of the combined group. As shown in Figure 3, two subgroups showed significantly improved OS (the group of patients with portal vein branch thrombosis without tumor extrahepatic metastasis) compared with patients with portal vein thrombosis and tumor extrahepatic metastasisas follows: HR, 0.34; 95%CI, 0.16–0.73; *P*  = 0.005; Statistical data for the group of patients with tumor extrahepatic metastasis without portal vein thrombosis are as follows: HR, 0.46; 95%CI, 0.23–0.91; *P*  = 0.027). However, combined therapy did not significantly improve OS of the group with main portal vein thrombosis without tumor extrahepatic metastasis (HR, 1.07; 95%CI 0.54–2.13; *P*  = 0.848).

In the combined group, patients were divided into four groups according to the extent of portal vein invasion and/or extrahepatic metastasis (Table 3). The median survival time of patients with main portal vein thrombosis (MPVT) without TEM was 5.8 months (Figure 3). Compared with patients with PVT and TEM, the OS of this group did not significantly improve (HR, 1.07; 95%CI, 0.54–2.13, *P* = 0.848). The median survival time of patients with portal vein branch thrombosis (PVBT) without TEM was 7.5 months. Compared with patients with PVT and TEM, the OS of this group was significantly improved (HR, 0.34; 95%CI, 0.16–0.73; *P* = 0.005).The median survival time was 8.0 months for patients with TEM without PVT. The OS of this group was significantly improved compared with patients with PVT and TEM (HR, 0.46; 95%CI, 0.23–0.91, *P* = 0.027). The median survival time was 3.5 months for patients with PVT and TEM.

**Table 3:** Subgroup analysis.

	Median survival time (months)	HR(95%CI)	P Value
1.Subgroup analysis in the combined group			
MPVT without TEM	5.8	1.07 (0.54–2.13)	0.848
PVBT without TEM	7.5	0.34 (0.16–0.73)	0.005
TEM without PVT	8.0	0.46 (0.23–0.91)	0.027
PVT with TEM	3.5	–	–
2.Subgroup analysis between combination and monotherapy group			
MPVT without TEM	5.8:4.7	0.56 (0.31–1.03)	0.064
PVBT without TEM	7.5:5.0	0.52 (0.29–0.95)	0.032
TEM without PVT	8.0:3.0	0.59 (0.36–0.98)	0.045
PVT with TEM	4.9:3.1	0.64 (0.33–1.24)	0.183

MPVT: main portal vein thrombosis; PVBT: portal vein branch thrombosis; TEM: extrahepatic metastasis; PVT: portal vein thrombosis.

### Subgroup Analysis of the Combined and Monotherapy Groups

**Figure 4:**
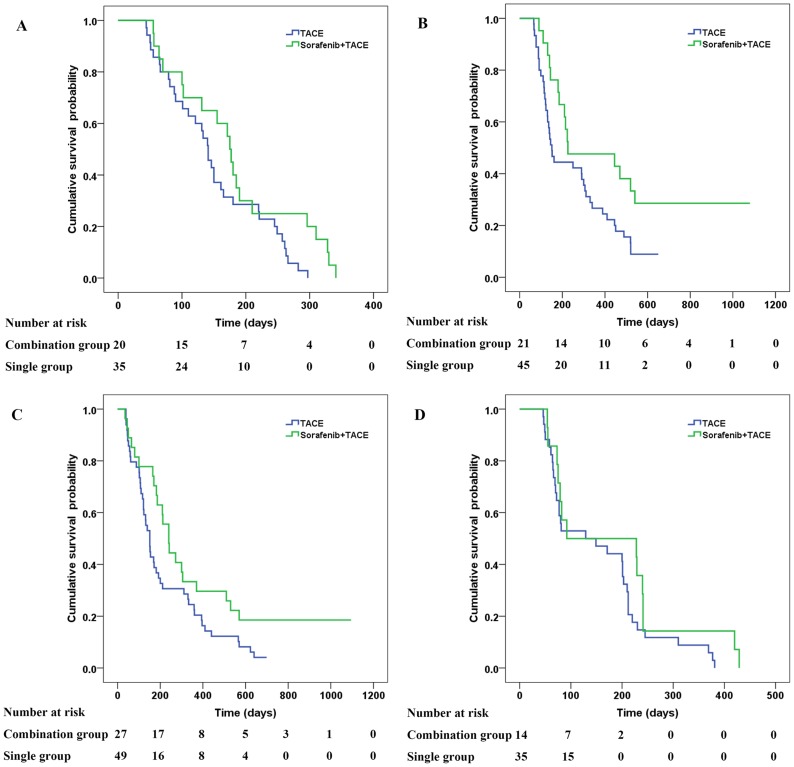
Kaplan–Meier analysis of OS of different subgroups of the combined and monotherapy groups. (A) Kaplan–Meier curves for OS of the combined and monotherapy groups for patients with main portal vein thrombosis (5.8 months vs. 4.7 months, respectively; *P* = 0.06). (B) Kaplan–Meier curves for OS of the combined and monotherapy groups for patients with portal vein branch thrombosis (7.5 months vs 5.0 months, respectively; *P* = 0.032). (C) Kaplan–Meier curves for OS of the combined and monotherapy groups for patients with tumor metastasis (8.0 months vs. 3.0 months, respectively; *P* = 0.045). (D) Kaplan–Meier curves for OS of the combined and monotherapy groups for patients with portal vein thrombosis and tumor metastasis (4.9 months vs. 3.1 months, respectively; *P* = 0.19).

The median survival time of patients with MPVT without TEM was 5.8 months and that of the monotherapy group was 4.7 months (*P* = 0.06, Figure 4A). There was no significant difference between two groups of patients (P>0.05) (HR, 0.56; 95%CI, 0.31–1.03; *P* = 0.064). The median survival time of patients with PVBT without TEM was 7.5 months and that of the monotherapy group was 5.0 months (*P* = 0.0029, Figure 4B). There was a significant difference between two groups of patients (*P*<0.05) (HR, 0.52; 95%CI, 0.29–0.95, *P* = 0.032).

The median survival time of patients with TEM without PVT was 8.0 months and that of monotherapy group was 3.0 months (*P* = 0.042, Figure 4C). There was a significant difference between two groups of patients (*P*<0.05) (HR, 0.59; 95%CI, 0.36–0.98, *P* = 0.045). The median survival time of patients with PVT and TEM was 4.9 months and that of the monotherapy group was 3.1 months (*P* = 0.183, Figure 4D). There was no significant difference between two groups of patients (P>0.05) (HR, 0.64; 95%CI, 0.33–1.24; *P* = 0.183).

### Time to Progression

**Figure 5:**
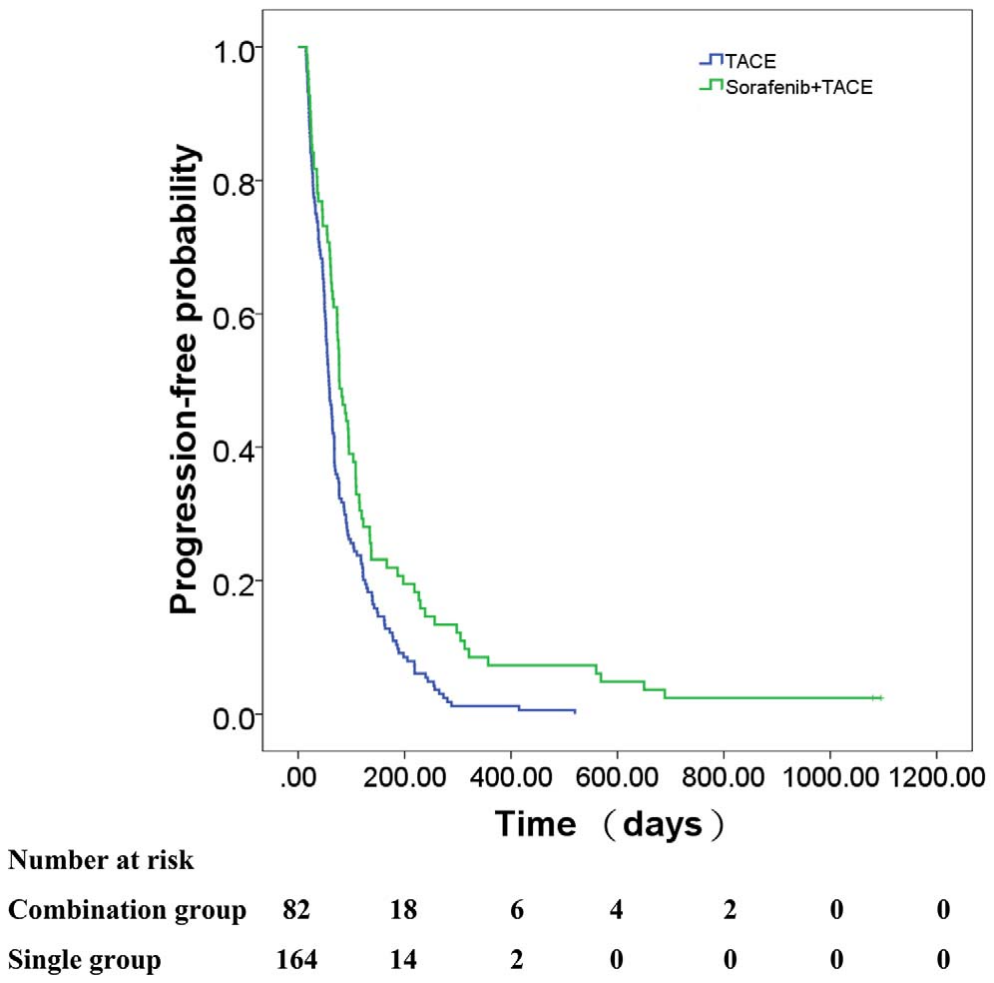
Kaplan–Meier analysis of TTP in the combined and monotherapy groups of propensity-matched patients. Median TTP of the combined group was significantly longer compared with the monotherapy group in the matched model (2.6 months vs 1.9 months, respectively; *P*  =  0.001).

During follow-up, tumors progressed radiologically in 244 of the 246 patients (99.2%) in the pooled cohort based on the mRECIST criteria (80 of 82 patients (97.6%) in the combined group and 164 of 164 patients (100%) in the monotherapy group. The median TTP was significantly longer in the combined group compared with the monotherapy group (2.6 months vs. 1.9 months, respectively; *P* = 0.001; Figure 5). The adjusted HR for TTP revealed an independent association with treatment regimen according to multivariate Cox analysis (HR, 0.62; 95%CI, 0.47–0.82; *P* = 0.001; Table 2. Ascites, Child–Pugh class, liver cirrhosis, and serum AFP level were other predictors of TTP (*P*<0.05 for each, Table 2).

### Serious Adverse Reactions (Grade 3 or 4 Toxicities)

Although we examined individual adverse events before each TACE session in the combined group, we did not include abnormal liver function and postembolization syndrome (nausea, vomiting, fever, and abdominal pain), because these usually occur shortly after TACE. Table 4 shows the sorafenib-induced adverse events in patients who received combined treatment. Grade 3 or 4 toxicities included hand-foot skin reaction (14.6%), diarrhea (6.1%), and hypertension (4.9%) as well as other severe adverse events, including gastrointestinal bleeding and hepatic encephalopathy in 2.4% and 3.7% of the patients, respectively.

**Table 4:** Grade 3/4 toxicities experienced by patients of the combined and monotherapy groups.

Adverse events	TACE plus sorafenib (%)	TACE alone(%)
Grade 3/4 adverse events		
Hand-foot skin reaction	12(14.6)	0(0)
Diarrhea	5(6.1)	0(0)
Hypertension	4(4.9)	0(0)
Severe adverse events		
Gastrointestinal bleeding	2(2.4)	7(4.3)
Hepatic encephalopathy	3(3.7)	7(4.3)

### Multivariate Subgroup Analyses of Survival

We performed stratified subgroup survival analyses according to the independent prognostic factors for OS identified using multivariate analysis. The combination of sorafenib with TACE provided significant benefits for OS in the subgroup of patients with PVBT without TEM (HR, 0.52; 95% CI, 0.29–0.95; *P* = 0.032), TEM without PVT (HR, 0.60; 95% CI, 0.36–0.99; *P* = 0.046), few ascites (HR, 0.61; 95% CI, 0.40–0.91; *P* = 0.016), Child–Pugh class A disease (HR, 0.64; 95% CI, 0.45–0.91; *P* = 0.012),and serum AFP>400 ng/mL (HR, 0.55; 95% CI, 0.39–0.77; *P* = 0.001).

## Discussion

In our present retrospective cohort study, we found that concurrent treatment with sorafenib and TACE might lengthen TTP compared with TACE monotherapy of patients with advanced HCC. This finding was substantiated by matched analyses based on stratified propensity scores and “traditional” multivariable Cox regression analysis. Moreover, after selective matching by propensity scores, a benefit of sorafenib plus TACE in prolonging OS in patients with HCC involving the portal vein or distant organ sites was demonstrated using a time-dependent Cox model. These statistical methods for controlling for confounders can yield extremely different estimates of treatment effects [19].

The potential benefits of TACE for treating patients with advanced HCC were revealed by several trials, even after sorafenib was universally established as first-line therapy for HCC [7], [20]–[22], [24], [25]. Recently, a large case-control study by Chung et al. indicates that TACE is a safe and effective treatment for patients who initially present with HCC with main portal vein invasion and that it may prolong their life expectancy compared with supportive care [8]. More recently, treating selected patients with BCLC stage C HCC achieved survival outcomes comparable with sorafenib treatment [23].

Sorafenib is the standard first-line treatment for patients with BCLC stage C HCC [15], [26]. In the SHARP and Oriental trials, monotherapy with sorafenib significantly prolonged OS (44% and 47%, respectively) and delayed TTP (73% and approximately 100%, respectively) in patients with advanced HCC compared with those who received placebo. However, sorafenib monotherapy confers fewer than 3 months of survival in Western and Asian populations [11], [12]. In a Korean cohort that received sorafenib monotherapy, median TTP and OS were 2.1 months and 5.9 months, respectively, which is similar to the results obtained in the Asia-Pacific phase III trial (2.8 months and 6.5 months, respectively).

In an attempt to compensate for the low cure rate obtained with TACE and its resultant hypoxia-induced angiogenic activity, TACE combined with sorafenib administration for unresectable HCC is being evaluated in one retrospective study [27] with promising outcomes and acceptable toxicity profiles. In the China trial, 67.1% of patients were diagnosed with BCLC stage C disease. However, the results of a phase III study (Sorafenib or Placebo in Combination with TACE) of Japanese and Korean patients were somewhat discouraging in terms of efficacy, but not the safety of sorafenib combined with TACE in patients with HCC restricted to BCLC stage B [28]. This result may be accounted for by the high percentages of sorafenib-treated patients who required interruptions (91%) and/or reduction (73%) of treatment, resulting in a much lower than planned median daily dose of sorafenib (386 mg).

Recently, a small cohort study conducted in the United States [29] indicates that combined therapy with TACE plus sorafenib is safe and equally effective as TACE monotherapy without unexpected adverse events; however, the small number of patients (13 patients in the combined group) may explain the outcome. In contrast, the significant benefit obtained in our study will encourage other researchers to challenge the results of studies specifically evaluating sorafenib in combination with TACE for treating patients with advanced stages of HCC. The results of randomized trials are similar to ours [12], [11].

In the subgroup analysis of the combined group, the median survival time was significantly different between patients with MPVT, those with PVBT, those with TEM, and those with both, indicating a poorer prognosis for patients with MPVT with/without TEM compared with the other two groups. In another subgroup analysis between the combined and monotherapy groups, we observed a significant difference in the median survival time of patients with PVBT or patients with TEM; however, no survival benefit was observed for patients with MPVT or patients with both.

We show here that combined therapy compared with TACE monotherapy did not effectively improve outcomes of HCC patients with MPVT with/without TEM. Moreover, tumor portal vein thrombosis further degraded liver function and exacerbated portal hypertension. Further, the main portal vein was totally occluded by tumor-induced thrombosis that blocked the supply of blood to normal liver tissue. Under these circumstances, sorafenib combined with TACE may instead significantly reduce blood supply to the liver, causing further ischemic liver impairment, which affects the prognosis. Accordingly, we suggest that the effects of combined therapy compared with monotherapy may differ depending on the extent of portal vein invasion. Therefore, our retrospective cohort study provides some new insights and guidance for treating patients with advanced HCC.

The present study has a number of strengths. TACE combined with sorafenib achieved improved outcomes compared with TACE monotherapy, and a higher overall survival rate was achieved for patients without main portal vein invasion. Further, this is a comparative study of a relatively large number of Chinese patients after treatment for advanced HCC.

Limitations of the present study include the inability to generalize the findings, because the study focused on patients of homogeneous ethnicity treated at a single institution. Furthermore, this was not a randomized, controlled clinical trial. Ideally, a phase III clinical trial is required that avoids selection bias, such as placebo effect and the choice of follow-up imaging platform (The choice of follow-up imaging platform may potentially allow discrepancies in time to progression if one group had more follow-up by MRI which may detect recurrences at a smaller size. However, it is difficult to accrue patients for such a study within a reasonable time. Under these circumstances, propensity score matching was used to mitigate the potential confounding (selection) bias of this nonrandomized trial. Other limitations include the reduced initial dose of sorafenib used to treat some patients as well as adjustment of dosages during treatment. However, according to a recent study, escalation of the dose of sorafenib may improve the patient's compliance and tolerance to prolonged therapy while not affecting efficacy or survival [30]. Finally, the physician's advice may significantly influence the patient's choice of therapy and therefore introduce selection bias.

In conclusion, this observational study aimed to minimize bias and approximate a randomized trial by basing the analysis on propensity score estimates. The results indicate that the addition of sorafenib to TACE therapy has a demonstrable effect in improving the median overall survival time of patients with advanced HCC. As a first or second-line therapy, TACE plus sorafenib may offer a promising survival advantage over sorafenib alone, particularly for treating patients with advanced HCC without main portal vein invasion. Further prospective randomized trials are required to substantiate our findings that combined therapy may represent an improvement of the current standard of care for advanced HCC.

## Supporting Information

Appendix S1 **The characteristics of excluded patients.**
(DOCX)
