# Supplementary material for: Sorafenib Combined with Transarterial Chemoembolization versus Transarterial Chemoembolization Alone for Advanced-Stage Hepatocellular Carcinoma: A Propensity Score Matching Study
Source: PLoS One. 2014 May 9;9(5):e96620. doi: 10.1371/journal.pone.0096620 (PMC4016022; doi:10.1371/journal.pone.0096620)
Supplement: Appendix S1 — The characteristics of excluded patients. (DOCX) [file pone.0096620.s001.docx]

**Appendix Supporting Information 1 (Appendix S1)**

**The characteristics of excluded patients**

41 patients were excluded from this study, the detailed reason as follow: no intrahepatic viable lesion (n=15)，liver transplantation (n=2)，lost to follow-up (n=14), other malignancy (n=10). In addition, 14 patients who were lost follow-up came from follow subgroups: main portal vein thrombosis (MPVT) without tumor extrahepatic metastasis (TEM) (n=4), portal vein branch thrombosis (PVBT) without TEM (n=3), TEM without portal vein thrombosis (PVT) (n=3), PVT and TEM (n=4).
